# Mechanical Behavior and Energy Dissipation of Woven and Warp-Knitted Pvc Membrane Materials under Multistage Cyclic Loading

**DOI:** 10.3390/polym14091666

**Published:** 2022-04-20

**Authors:** Shanshan Guo, Linlin Wang, Guangwei Shao, Huiqi Shao, Jinhua Jiang, Nanliang Chen

**Affiliations:** 1 Shanghai Collaborative Innovation Center of High Performance Fibers and Composites, College of Textiles, Donghua University, Shanghai 201620, China; guoss@dhu.edu.cn (S.G.); 15821989252@163.com (L.W.); shaogw@dhu.edu.cn (G.S.); 2 Engineering Research Center of Technical Textiles, Ministry of Education, Donghua University, Shanghai 201620, China; hqshao@dhu.edu.cn; 3 Innovation Center for Textile Science and Technology, Donghua University, Shanghai 201620, China

**Keywords:** woven and warp-knitted PVC membrane materials, multistage cyclic loading, energy dissipation, damage variable

## Abstract

In order to study the mechanical behavior and energy dissipation of architectural membrane materials under multistage cyclic loading, the deformation behavior, energy dissipation, and damage characteristics of four kinds of warp-knitted and woven polyvinyl chloride (PVC) membrane materials were analyzed using multistage cyclic loading experiments. The results show that, compared with the uniaxial tensile strength, the peak values of the cyclic loading and unloading of the four material samples are lower in the warp direction but higher in the fill (weft) direction. Under multistage cyclic loading, the loading and unloading moduli of the warp knitting membrane increase with the increase in fabric density. At the same fabric density, the loading modulus and the unloading modulus are smaller than those of the warp knitting material. The total absorbed strain energy, elastic strain energy, and dissipation energy of the fill samples are higher than those of the warp samples at a low load level but lower than those at a high load level. PVC membrane materials’ use strength should be controlled below a 15% stress level under long-term external force loading. In the cyclic loading process, the four PVC membrane materials are viscoelastic–plastic, so it is reasonable to define the damage variable based on the accumulation of plastic deformation.

## 1. Introduction

Flexible composites are generally made of two or more components with different thermal or mechanical properties and show great variations and complexity in tensile behavior compared to conventional material systems. Composite fabric reinforcements have a myriad of applications, including in the aerospace, automotive, and transportation areas [1,2]. Warp-knitted biaxial and woven fabric-reinforced membrane materials are two of the most common types of flexible composites [3]. Their applications include rotor blades, wind turbines, yachts, and bridge deck slabs [4,5]. Due to the complexity of the microstructure of the internally reinforced fabric, the nonlinearity of the material and geometry, and the difference in the control of the warp and fill (weft) tension in the process of weaving and coating, membrane materials exhibit different macroscopic mechanical behaviors under different applied loads [6,7,8,9]. In the process of use, the material is subjected to repeated effects, such as gusts, rain, and snow, and repeated adjustments of external load, and the membrane material is usually in a state of repeated loading and unloading [10,11]. In addition, the deformation, strength characteristics, and fracture damage mechanical properties of membrane materials are closely related to the stress state and loading history. At the same time, the failure of membrane materials under different stress conditions is actually the macroscopic manifestation of the fracture and expansion of internal micro-cracks and defects under loading conditions. Therefore, in order to prevent the mechanical failure of membrane structure materials under repeated loading and unloading, it is necessary to analyze their mechanical behavior under multistage cyclic loading.

Studies have found that the elastic modulus and residual deformation of polytetrafluoroethylene membrane materials, vinylidene fluoride/polyethersulfone membrane materials, and woven fabric-reinforced flexible composites are closely related to the number of repeated loading and unloading cycles [12,13]. It was found that there is a coupling relationship between the warp and fill stress ratio and the elastic modulus, and the influence law of the warp and fill stress ratio and the coupling elastic modulus was also analyzed [14,15]. The uniaxial and biaxial tensile behaviors of PTFE membrane materials under cyclic loading are mainly affected by the stress amplitude, temperature, and reinforced skeleton fabric structure, and the loading history has an obvious influence on the mechanical behavior [16,17,18].

Compared with a composite material with a woven structure, the warp-knitted biaxial structure has been used more and more as a reinforcement for flexible membrane composites due to the biaxial characteristics of the fiber arrangement direction [19,20,21]. Moreover, the most common problem of polyester industrial yarns, such as lightbox fabrics, in their process of use [22] is mechanical failure. It was found that, while the coated biaxial warp-knitted fabric demonstrates anisotropic properties under monoaxial tensile conditions, the same fabric behaves more isotropically under multi-axial tensile loads [23]. A three-dimensional model of woven composites considering the viscoelastic nature of a matrix was tested to gain a better understanding of fabric composites [24,25]. Thus, it is important to question which one has the best mechanical behavior under multistage cyclic loading. It is necessary to study and compare the mechanical differences between warp-knitted PVC membrane materials and woven fabric-reinforced PVC membrane materials systematically. However, the above studies mainly focus on the change in the elastic modulus and linearity of the tensile curve. The energy dissipation and evolution in multistage cyclic loading processes are aspects that have been less studied.

The aim of this work is to investigate the mechanical properties of woven and warp-knitted PVC membrane materials under multistage cyclic loading. This paper focuses on the peak strength of the cyclic loading and deformation characteristics, the mechanics of elasticity, and fracture damage mechanic performance characteristics in order to compare the performances of the woven and warp-knitted PVC membrane materials. The advantages and disadvantages of the linear fitting and direct equivalent simulation are compared to calculate the loading and unloading moduli. Additionally, the macroscopic mechanical behavior, energy dissipation, and damage evolution were analyzed in order to further comprehend the behavior of the damage mechanics of the woven and warp-knitted PVC membrane materials. The knowledge obtained from these tests provides a better understanding of the performance and application of dynamically loaded flexible composites. It also provides a reference for their engineering application.

## 2. Materials and Methods

### 2.1. Materials

Two flexible composites were selected as samples, and they were enhanced using polyester biaxial warp-knitted and woven fabrics (Shanghai Shenda Kobond New Material Co., Ltd., China). The warp and fill lining/yarns were 1000D/192F (111.11Tex) high-strength polyester industrial yarns. The specification of the binding yarn in the biaxial warp-knitted fabric was 75D/25F(83.33Tex) DTY polyethylene terephthalate (PET). Two types of fabric were bonded with a PVC membrane on both sides by the coating. The first type was a biaxial warp-knitted fabric with a tricot binding stitch, and the second type was a woven fabric with a plain weave construction.

All samples were laminated with the same pure PVC membrane, and the coating thickness of each sample was the same. The 3 warp/weft laying yarn densities of the biaxial warp-knitted fabrics were 9 wales/inch and 9 courses/inch, 12 wales/inch and 12 courses/inch, and 18 wales/inch and 18 courses/inch. However, 1 fabric count (18 × 18 picks/inch) was selected to prepare the woven fabrics as controls. The specifications of the samples are listed in Table 1.

### 2.2. Sample Preparation and Experimental Methods

The width of the samples was (50 ± 0.5) mm, the length was (300 ± 0.5) mm, and the effective clamping distance was (200 ± 0.5) mm.

In the multistage cyclic loading experiment, the automatic loading and unloading rate was 5 mm/min. There were 5 cyclic peak stress levels (100 times per level), which were 5%, 10%, 15%, 20%, and 25% of the samples’ fracture strength. The samples were named K_1_, K_2_, K_3_, and W_1_. In order to ensure that the samples were always in a state of tension during the experiment, the lower limit of unloading stress was the pre-tension level before the first cycle.

In order to compare the strength characteristics of the PVC membrane materials under multistage cyclic loading, a uniaxial direct tensile fracture test was conducted on the warp and fill directions of samples; the loading speed was set at 10 mm/min, and these samples were named K_1′_, K_2′_, K_3′_, and W_1′_. All the experiments were carried out on the WDW-20 Hualong strength tester model at room temperature.

## 3. Results and Discussion

### 3.1. Analysis of Relaxation Characteristics

**Figure 1:**
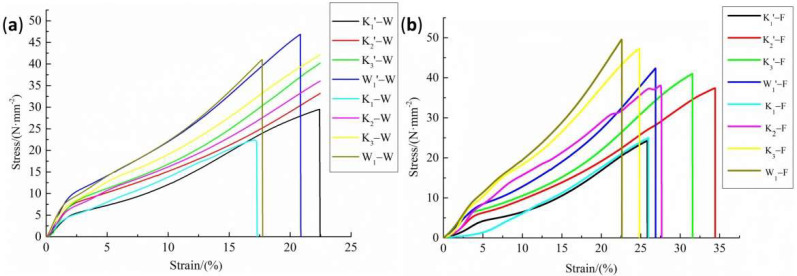
Fracture stress–strain curves of the tested specimens. (**a**) Warp direction; (**b**) fill direction.

**Figure 2:**
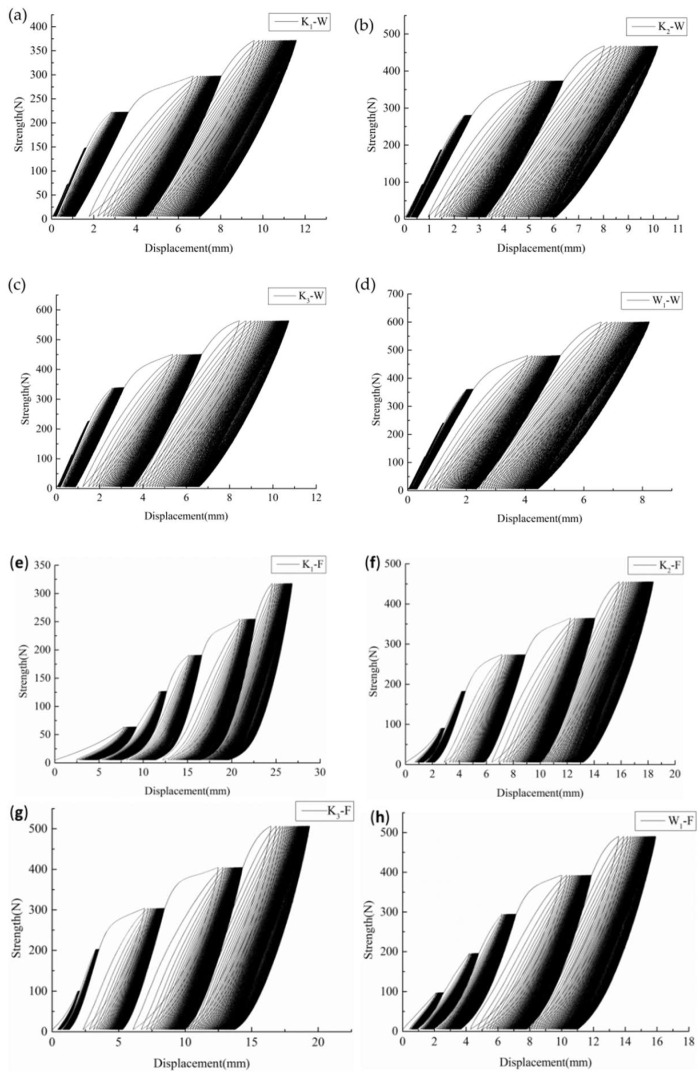
Stress–strain curves of the tested specimens under multistage cyclic loading and unloading. (**a**–**d**) Warp direction of K_1_, K_2_, K_3_, and W_1_; (**e**–**h**) fill direction of K_1_, K_2_, K_3_, and W_1_.

The stress–strain curves of PVC membrane materials under direct stretching and after multistage cyclic loading are shown in Figure 1 and Figure 2. It is shown in Figure 1 that the stress drops suddenly after the peak strength in the direct tensile curves of both the warp and fill direction samples of membrane materials. While under multistage cyclic loading, the stress–strain curves change periodically. Figure 2 shows that the areas of hysteresis loop formed by the loading and unloading curves increase with the increase in the number of cycles. The results show that the direct warp tensile fracture strengths are 29.51 N/mm^2^ (K_1′_), 40.03 N/mm^2^ (K_2′_), 46.73 N/mm^2^ (K_3′_), and 46.88 N/mm^2^ (W_1′_), and the average peak strengths of the multistage cyclic fracture are 22.39 N/mm^2^ (K_1_), 38.68 N/mm^2^ (K_2_), 45.19 N/mm^2^ (K_3_), and 40.99 N/mm^2^ (W_1_), which are 3.27–24.12% lower than the direct tensile fracture strength. The fill uniaxial tensile fracture strengths are 24.27 N/mm^2^ (K_1′_), 37.42 N/mm^2^ (K_2′_), 41.00 N/mm^2^ (K_3′_), and 42.35 N/mm^2^ (W_1′_), and the average peak tensile strengths of the multistage cycle are 24.97 N/mm^2^ (K_1_), 38.05 N/mm^2^ (K_2_), 47.29 N/mm^2^ (K_3_), and 49.55 N/mm^2^ (W_1_), which are 1.7–17% higher than the original tensile fracture strength.

The fabric structure is greatly affected by the weaving and coating process; thus, in order to ensure a clear shed during the weaving process, the warp yarns were subjected to a higher tension and better elongation. In the process of coating, the warp yarns were also subjected to a higher tension, so they were further straightened and the degree of buckling was reduced. Under the action of the applied load, the warp strength utilization rate was higher, resulting in a higher direct tensile strength of the membrane materials [6].

In the process of weaving and coating, the warp yarns were subjected to high tension and repeated tensile, bending, friction, and impact stresses, and the macromolecules in the fibers were fully straightened. During the multistage cyclic loading process, slips occur between macromolecules, resulting in a decrease in the intermolecular forces. Macroscopically, the peak strength of multistage cyclic loading is lower than the direct tensile fracture strength [26]. However, the fill yarns were subjected to a low level of tension and the tension fluctuation was small in the weaving and coating process; cyclic loading is beneficial as it further straightens the macromolecules in the fiber and enhances the effect of bearing the applied load, which is macroscopically manifested since the multistage cyclic peak strength is higher than the direct tensile fracture strength.

It can be seen from Figure 2 that, under five different stress gradients, the strain produced by materials with a high density is lower than that produced by materials with a low density in both the warp and fill samples, which shows K_3_ < K_2_ < K_1_, while the strain produced by warp knitting and woven materials with the same density is W_1_< K_3_ in comparison.

The reason for this is that, according to the structural characteristics of the membrane material, the PVC resin coating on the surface of the membrane material is bonded to the surface of the base fabric by an adhesive, and the resin coatings on both sides are bonded to each other in the gap between the warp and the fill yarns. The PVC resin on both sides of the yarn interweaving point is bonded to the yarn in the base fabric. Due to the different adhesion of the interface, the void ratio between the high-density K_3_ yarns is lower. Therefore, the viscoelastic deformation caused by the PVC resin on the surface of the K_1_ and K_2_ materials is more involved, resulting in a larger shape variation under the same stress level. Similarly, compared with the binding yarn gap caused by the warp knitting structure of K_3_, the yarns are more closely interwoven in the woven structure of W_1_, so the shape variable of W_1_ is smaller.

### 3.2. Analysis of the Cyclic Elastic Modulus

**Figure 3:**
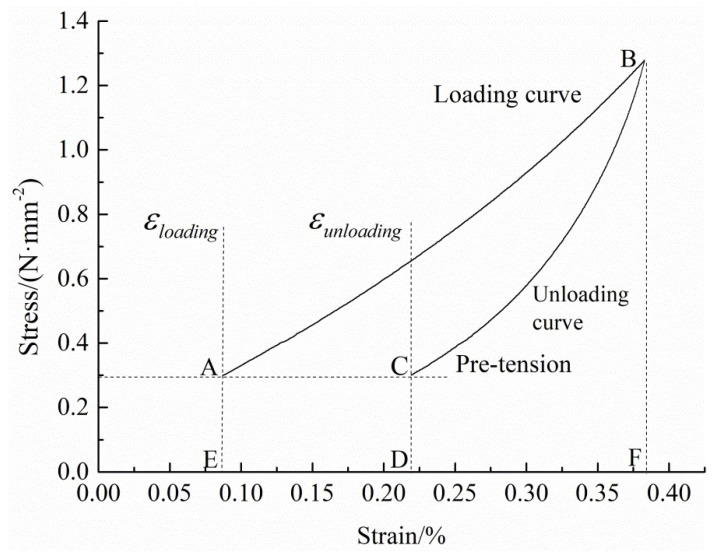
Level 2 stress–strain curves of the K_1_ warp specimen.

In the cyclic loading process, two main methods are used for calculating the elastic modulus of the loading and unloading: the linear fitting method and the approximate imitation method. In the linear fitting method, based on the initial stage of the loading and unloading stress–strain curves, the elastic modulus of loading and unloading can be obtained by linear fitting. This method can reflect the characteristics of the elastic modulus of materials. In the approximate imitation method, based on the slope of the connecting line of the initial loading point A and loading terminal B used to calculate the loading elastic modulus, and based on the slope of the connecting line of the initial unloading point B (same with loading terminal) and unloading terminal C used to calculate the unloading elastic modulus, the key step is to approximate the load and unload curves to a straight line. The slope of the straight line is the loading or unloading modulus of elasticity, which is shown in Figure 3.

The multistage cyclic loading and unloading curves of both the fill and warp samples show obvious nonlinear characteristics. When calculating the elastic modulus of the loading and unloading, the slope of the line between points A and B is higher than that of the initial section of the curve AB, and the slope of the line between points B and C is also higher than that of the initial section of the curve BC. Compared with the linear fitting method, the elastic modulus of the loading and unloading calculated based on the approximate imitation method is higher and cannot objectively reflect the mechanical behavior of the materials.

**Figure 4:**
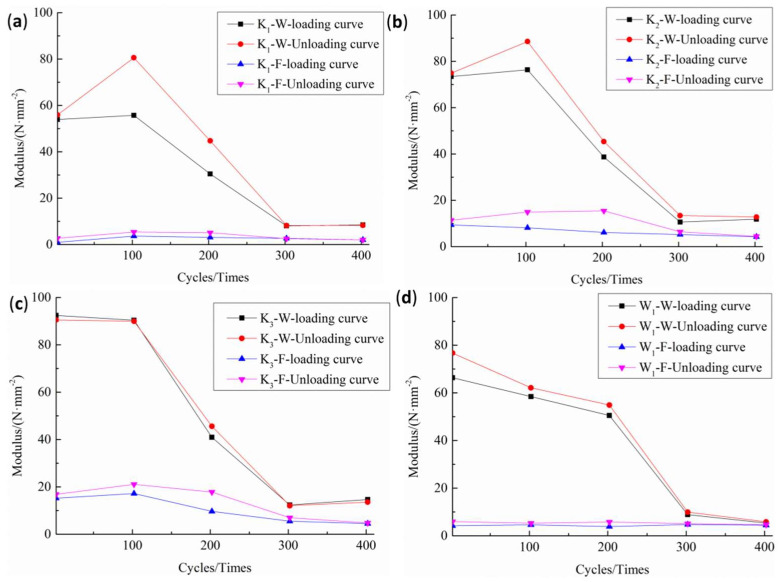
Elastic modulus curves: (**a**) warp and fill specimens of K_1_; (**b**) warp and fill specimens of K_2_; (**c**) warp and fill specimens of K_3_; and (**d**) warp and fill specimens of W_1_.

Therefore, in this paper, the elastic moduli of the loading and unloading were calculated based on the linear fitting method, and their curves are shown in Figure 4. It can be seen that the loading and unloading elasticity of the PVC membrane materials decreases with an increase in the number of cycles, while the loading and unloading elasticities of the K_1_ and K_2_ fill samples increase slightly with an increase in the number of cycles and those of the K_3_ and W_1_ decrease slightly. In the same cycle, the unloading modulus is higher than the loading modulus, and the difference between them first increases and then decreases with the increase in the number of cycles.

By comparing the factors that affect density, the values of the loading and unloading moduli increase with the increase in density, the order of which is K_3_ > K_2_ > K_1_. At the same density, the loading and unloading moduli of the woven material are lower than those of the warp knitting material (W_1_ < K_3_). Under cyclic loading, the macromolecular chain of the reinforcing fiber and coating materials with a low adhesion slips first and provides a new combination with each other. As the macromolecular chain becomes straighter, the stresses improve, and the fiber deformation is mainly caused by the extension and retraction of the macromolecular chain. The aspect of macro performance evaluated is the modulus of elasticity increased.

### 3.3. The Characteristics of Energy Dissipation

It is shown in Figure 3 that the area of ABFEA under curve AB is the total absorbed strain energy, which represents the work done by external force on the sample during a loading and unloading cycle. The CBFDC area under the curve BC is the elastic strain energy of the cycle. The area of ABCDEA is dissipated energy, which is used for sample damage and plastic deformation. The relationship between the total absorbed strain energy, elastic strain energy, and dissipated energy can be expressed as follows:

(1) $$U_{d}=U-Ue$$

where *U* is the total strain energy, mJ/mm^3^; *Ue* is the elastic strain energy, mJ/mm^3^; and *U_d_* is the dissipated energy, mJ/mm^3^.

The loading and unloading curves show hysteresis rather than coincidence loops, which is mainly due to the fact that PVC membranes are typical viscoelastic materials. During multistage cyclic loading, the material produces plastic deformation, so the hysteresis ring is not closed.

**Figure 5:**
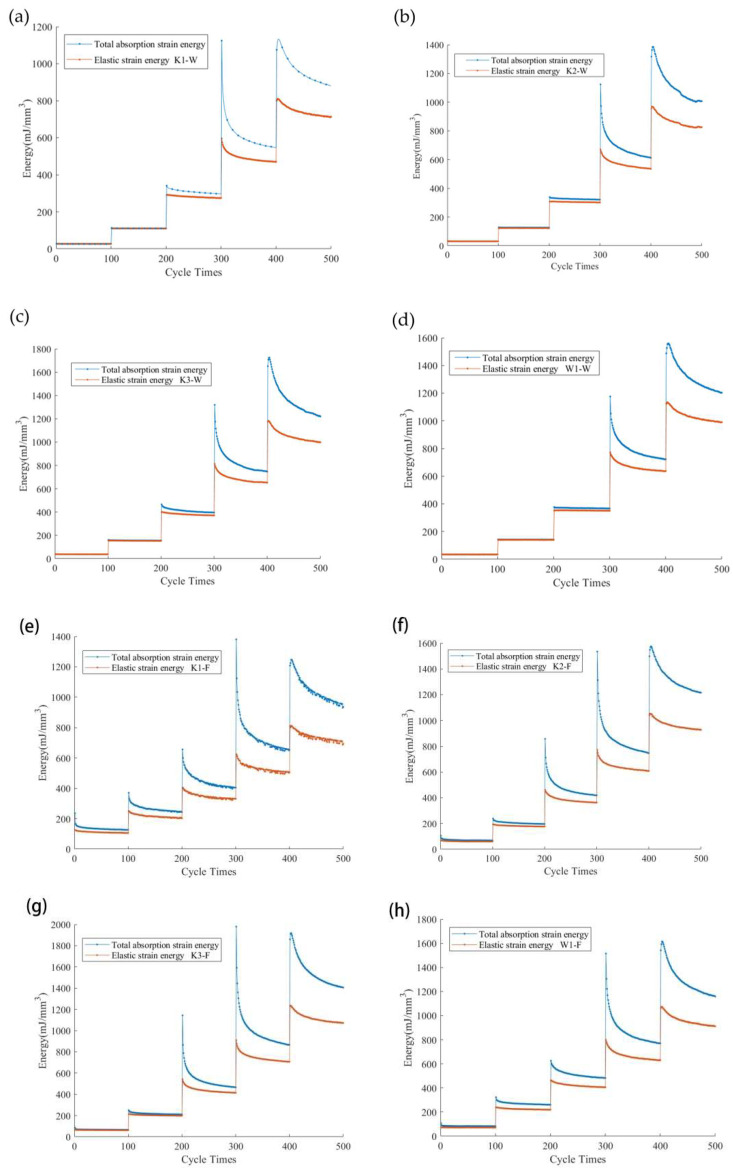

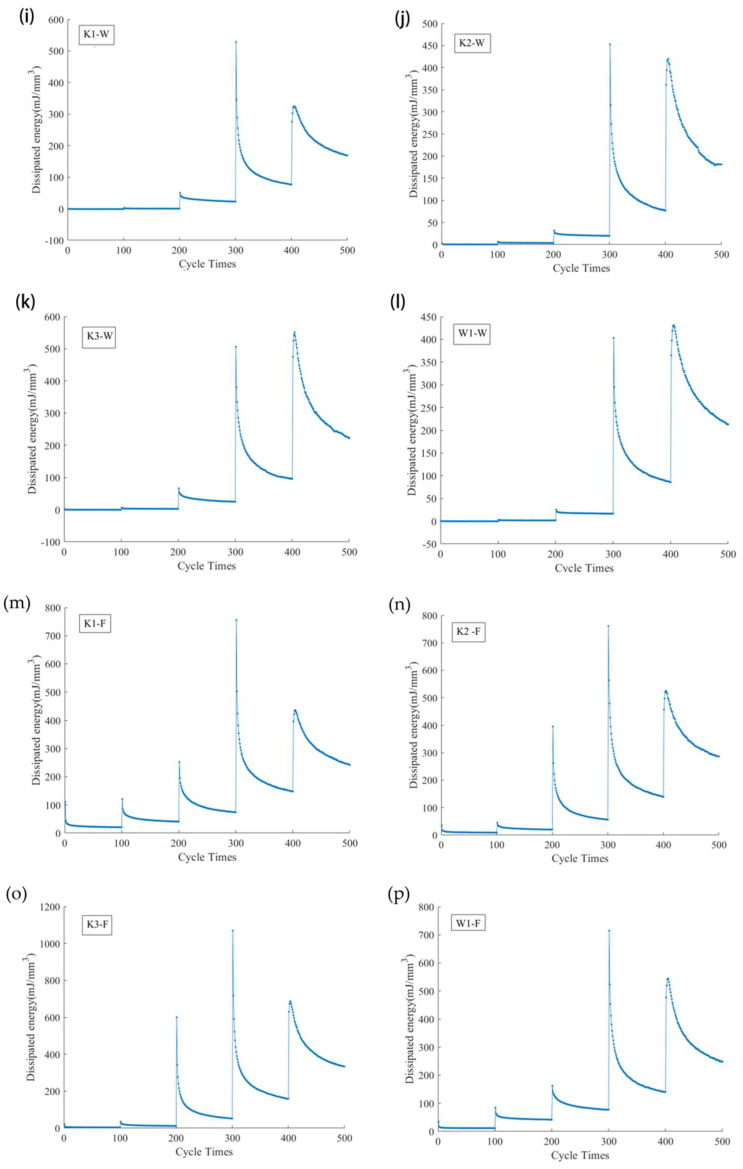
Energy variation curves with stress. (**a**–**d**) Total absorption strain energy and elastic strain energy of the warp direction of K_1_, K_2_, K_3_, and W_1_. (**e**–**h**) Total absorption strain energy and elastic strain energy of the fill direction of K_1_, K_2_, K_3_, and W_1_. (**i**–**l**) Dissipated energy of the warp direction of K_1_, K_2_, K_3_, and W_1_. (**m**–**p**) Dissipated energy of the fill direction of K_1_, K_2_, K_3_, and W_1_.

The variation trends of the total absorbed strain energy, elastic strain energy, and dissipated energy of samples are shown in Figure 5. It can be seen that the energy variation curves of the warp and fill samples are similar and show a nonlinear growth trend, indicating that the energy absorption, storage, and dissipation mechanisms of the warp and fill samples are consistent under multistage cyclic loading.

**Figure 6:**
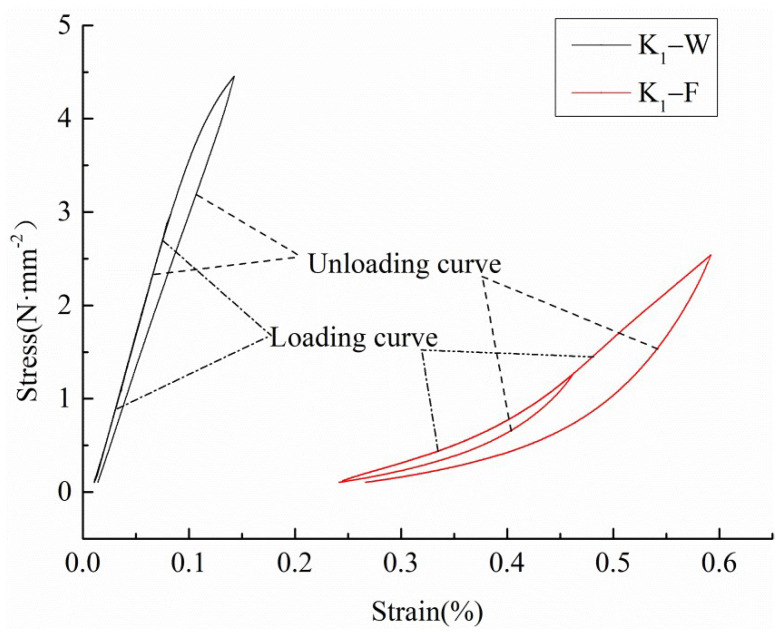
Second and third level stress–strain curves of the tested K_1_ samples.

The total absorbed strain energy, elastic strain energy, and dissipated energy of the weft samples are lower when the cyclic load is the same. Taking the warp and fill samples of K1 for the second loading cycle as an example (Figure 6), due to the high loading and unloading moduli of the warp sample, the initial loading and unloading strains of the warp sample are small when they reach the same cyclic load peak, so the area under the loading and unloading curve is smaller. That is, the total absorbed strain energy and elastic strain energy are smaller. Meanwhile, in the same circular process, the dissipation energy of the warp sample is lower under the double effects of the difference between loading and unloading moduli and plastic deformation because the plastic deformation of this sample is smaller [27].

Figure 5 indicates that, under the experimental conditions in this paper (5%, 10%, 15%, 20%, and 25% of the samples’ fracture strength), when the cyclic peak load level is low, the total absorbed strain energy, elastic strain energy, and dissipated energy of the weft samples are relatively higher.

It can be seen from Figure 5a,b that the total absorption strain energy and elastic strain energy of the K_1_, K_2_, K_3_, and W_1_ samples have the same variation rule; they all increase with the increase in density, and the fill direction value is larger than the warp direction. The comparison of woven and warp-knitted samples of the same density shows K_3_ > W_1_. Figure 5c,d indicates that the overall trend of the dissipated energy of the K_1_, K_2_, K_3_, and W_1_ samples is relatively consistent, increasing with the increase in density, and, furthermore, the fill direction value is larger than the warp direction. The comparison of woven and warp-knitted samples of the same density shows K_3_ < W_1_.

When the cyclic load peak level is low, the energy absorption and storage are mainly realized by the change of fabric structure and macromolecular conformation of the yarn and coating materials. The energy dissipation mainly comes from the slip of the macromolecular chains in the reinforcement fiber and coating material, the extension of the original damage and the destruction of the binding point between the coating and the reinforcement fiber.

Due to the difference in strain in the manufacturing process of weaving and coating, relative to the warp yarns, the fill yarns’ macromolecular chain segments are in lower degrees, and the yarns’ extended degrees are insufficient. Under the applied load, the elongation of the fill yarns is mainly caused by a decrease in buckling and the macromolecular chain segments slipping; the fill elongations result in a lower modulus, so the fill samples may show a higher total absorption. In the recovery stage, the stretched macromolecular chain segments return to their initial state, the fill yarns have a degree of buckling, and their capacity for energy storage is stronger.

In the cyclic loading process, the original weak areas are destroyed, and due to the great change in the fills’ buckling structure, the probability of failure of the bonding points between the reinforcement fibers and the coating materials is higher and is macroscopically expressed as the increase in dissipated energy.

When the cyclic load peak level is high, the absorption, storage, and dissipation of energy are mainly dependent on the stretching, recovery, and slip of the macromolecular chains in the reinforced fiber and coating materials. The higher the load ratio is, the higher the degree of elongation of the macromolecular chain segment and the greater the degree of slip will be, while the recovery capacity will also be improved.

### 3.4. The Rule of Rapid Elastic Recovery Rate

The ratio of the elastic strain energy *Ue* to the total absorbed strain energy *U* is defined as the energy rapid elastic recovery rate *u*, as follows:

(2) $$u=\frac{Ue}{U}\times 100\%$$

**Figure 7:**
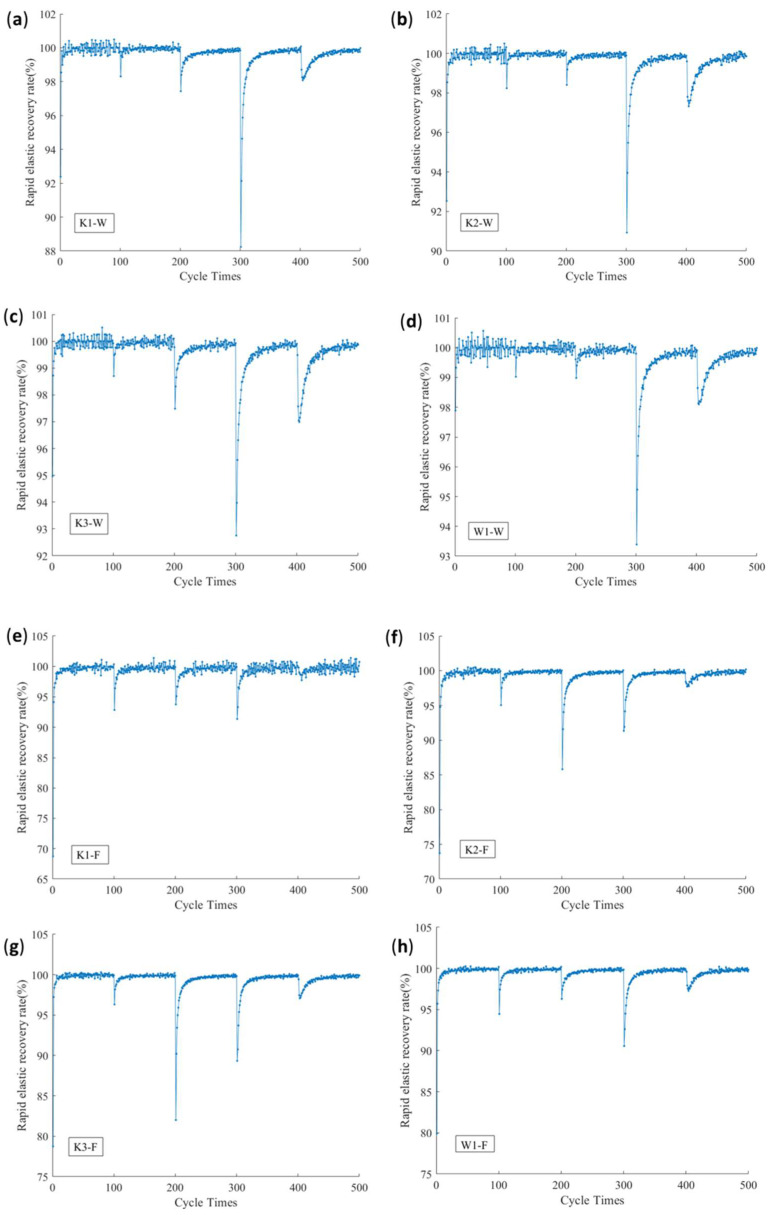
Variation curves of the rapid elastic recovery rate with stress. (**a**–**d**) Rapid elastic recovery rate of the warp direction of K_1_, K_2_, K_3_, and W_1_. (**e**–**h**) Rapid elastic recovery rate of the fill direction of K_1_, K_2_, K_3_, and W_1_.

Figure 7 shows the curves of the energy rapid elastic recovery rate. It can be seen that, as the peak of the multistage cyclic load increases, the energy rapid elastic recovery rates of the fill and warp samples show a nonlinear change. The warp samples’ *u* value of K_1_, K_2_, K_3_, and W_1_ tend towards 100% during the first 300 instances of multistage cyclic loading, and the fill samples’ *u* value of K_1_, K_2_, K_3_, and W_1_ tend towards 100% during the first 200 instances of multistage cyclic loading. When the warp samples are loaded at a 20% (400 cycles) stress level of the fracture strength of the sample, the *u* value decreases significantly, to 88% (K_1_), 91% (K_2_), 92.5% (K_3_), and 93.2% (W_1_). There was an overall trend of increase with the increase in density. When the fill samples of K_2_ and K_3_ are loaded at a 15% stress level (300 cycles), the u value decreases significantly to 85.6% (K_2_) and 82.5% (K_3_), while the u value of the fill samples of K1 and W1 decreases significantly when the samples are loaded at 20% stress level (400 cycles) and the rapid elastic recovery rate decreases to 91.5% (K1) and 90.8% (W1), respectively. The rapid elastic recovery rate of the four samples is higher in the fill direction than in the weft direction. The resilience of W_1_ is better than that of K_3_.

To explore the causes of this phenomenon, at the beginning of the multistage cyclic load, the proportion of dissipated energy *U_d_* is low, and most of the absorbed energy turns into elastic strain energy *Ue*. This shows that the energy consumption, which is caused by the expansion of the internal defects of the slip of the materials, fibers, and coating materials in the debonding of the macromolecular chain, fibers, and coating materials, is relatively small. It is mainly turned into the recoverable elastic strain energy *Ue*. With the increase in the multistage cyclic load peak, the dissipated energy *U_d_* increases, while the energy dissipation rate increases and reaches the maximum value when the cyclic load is in the range of a 15–20% stress level of the fracture strength. When the peak value of the cyclic load exceeds this critical value, although the dissipated energy continues to increase (Figure 5), most of the energy is converted into elastic strain energy *Ue*, and the energy dissipation rate presents a trend of gradual decrease. From the perspective of the energy dissipation rate, PVC membrane materials’ use strength should be controlled below a 15% stress level under long-term external force loading.

It can also be seen from Figure 7 that the rapid elastic recovery rate of the fill sample is relatively low in the first stage of the cyclic loading and unloading process, which is still related to the difference in the tension state of the warp and fill direction during weaving and coating processing.

### 3.5. Definition and Analysis of Damage Variables

Lemaitre et al. [28] defined the damage variable from the perspective of the change of elastic modulus, that is, the elastic modulus is taken as the damage factor, and the ratio of the elastic modulus of the damaged sample to that of the non-destructive sample can be used to define the damage variable *D_E_*:

(3) $$D_{E}=1-E\prime /E$$

where *E* and *E*’ are the elastic moduli of undamaged material and damaged materials, respectively, in N/mm^2^.

Damage is reflected in the generation and expansion of material defects, during which the elastic modulus gradually decreases. When the material is completely damaged, the material is also completely destroyed, and at this time, the elastic modulus *E*’ is 0. The maximum damage variable of the material is 1.0.

Figure 5 shows that, during the multistage cyclic loading, the elastic modulus of the PVC membrane material presents an increasing trend, so it inevitably obtains the negative damage variable based on the elastic modulus change, and the material is thus characterized as “negative damage”. The damage variable’s value and the actual damage evolution characteristics are contradictory, so in multistage cyclic loading conditions, it is unreasonable to use the elastic modulus as the damage factor.

There are two types of failure limits that may occur during cyclic loading. (1) When the material is under the action of cyclic loading, although there is no plastic deformation, actually the internal defects are produced. When the damage accumulation reaches a certain level, the damage in the material structure accumulates, and there is a failure in the material structural fatigue. (2) When the material is in an elastic–plastic working state, cyclic loading causes plastic deformation and also causes damage to the material. When the accumulated plastic deformation exceeds a certain limit, the service performance of the material is weakened until the failure limit appears.

From the viewpoint of energy conversion, the accumulation of damage represents the energy dissipation, which can be defined as the damage variables from the perspective of energy dissipation. So, the division of the dissipation of energy, which is accumulated in the process of each cyclic loading to the final loading cycle dissipation, with the total strain energy results in the damage variable *D*u, during cyclic loading to the level i. The damage variable *D*u (*i*) can be represented as:

(4) $$D_{U}(i)=\sum_{k=1}^{i}\frac{U_{d}^{k}}{U(t)}$$

where *U*(*t*) is the total strain energy of the last stage cycle, in mJ/mm^3^.

**Figure 8:**
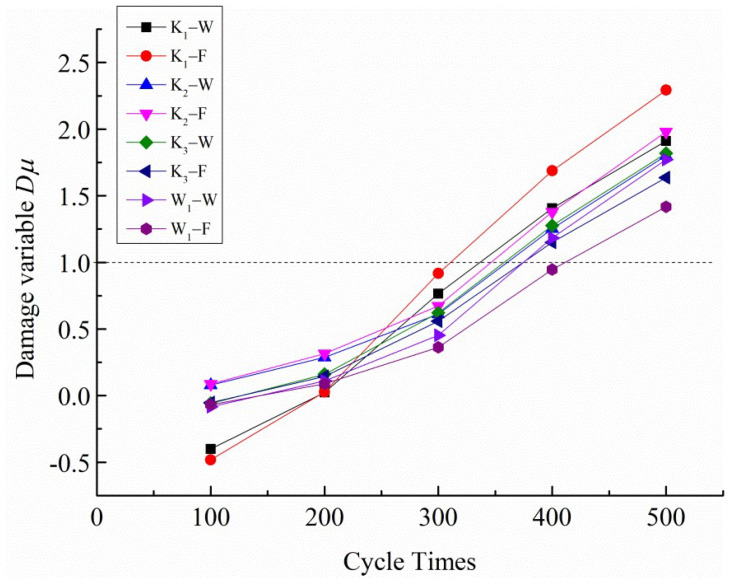
Curves of the damage variable *D*u and cycle number.

The relationship between the calculated damage variables and cyclic progression based on Equation (4) is shown in Figure 8. It can be seen that, when the number of cyclic loading instances exceeds about 400, the damage variable values of both the warp and fill samples are greater than 1.0, which is inconsistent with the damage limit of 1.0. Therefore, Equation (4) cannot be used to calculate the damage variable.

From Figure 2, Figure 3 and Figure 6, it can be observed that the PVC membrane materials’ loading and unloading curves do not overlap and the hysteresis loops are not closed, which shows that, in the process of multistage cyclic loading, the PVC membrane materials are in an elastoplastic state, and cyclic loading will cause the accumulation of plastic deformation, resulting in the degradation of the properties of the material. Based on this, the damage variable can be defined as the ratio of plastic strain accumulated in each loading cycle to the fracture strain of the non-destructive sample (the original sample). So, the damage variable *D*ε(i) can be expressed as:

(5) $$D\varepsilon (i)=\frac{\sum_{k=1}^{i}\varepsilon_{per}(k)}{\varepsilon_{b}}=\frac{\sum_{k=1}^{i}[\varepsilon_{unloading}(k)-\varepsilon_{loading}(k)]}{\varepsilon b}$$

where *ε_b_* is the fracture strain of the specimen under unidirectional tension, in mm/mm, and *ε_per_* is the plastic deformation produced by each cycle, in mm/mm.

**Figure 9:**
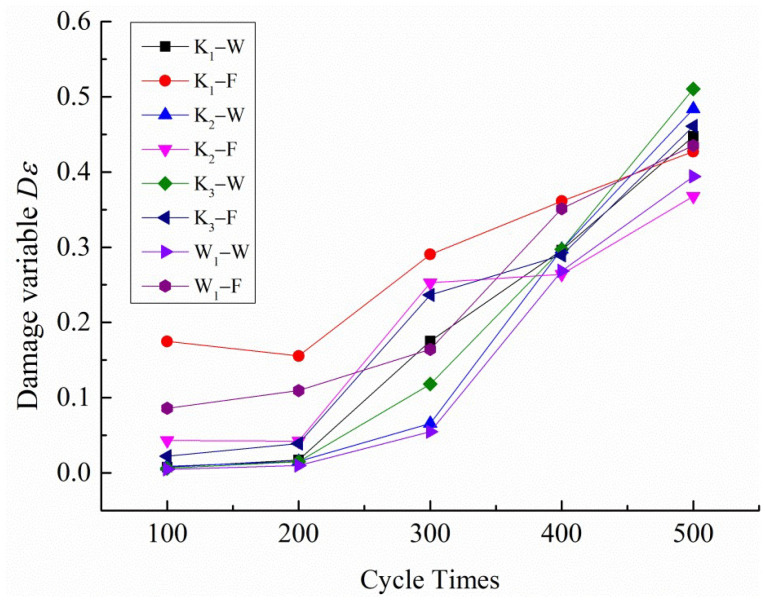
Curves of the damage variable *Dε* and cycle number.

The variation relationship between the damage variable *Dε*, which is calculated based on Equation (5), and the number of cyclic loading instances is shown in Figure 9.

It can be seen from Figure 9 that the *Dε* value of the damage variable, calculated based on the plastic deformation accumulation, is between 0 and 1; the four samples K_1_, K_2_, K_3_, and W_1_ satisfy this rule. So, within the range of the damage limit, it can be considered that it is reasonable to define the damage variable based on the plastic deformation accumulation [29]. At the same time, it was found that, during multistage cyclic loading, the damage of both the fill and warp samples showed a similar nonlinear growth trend, indicating that the damage mechanism of the PVC membrane material is similar in the fill and warp directions, and the damage level of the fill samples is higher than that of the warp samples under the same cyclic load peak conditions. In addition, the damage level of K_3_ is higher than that of W_1_ from the difference in the fabric structure between the woven and warp-knitted fabrics.

## 4. Conclusions

In this paper, four kinds of PVC membrane materials were tested and analyzed under multistage circulation. The deformation behavior and mechanism of warp-knitted and woven PVC membrane materials under multistage cyclic loading were studied and analyzed, especially in terms of the multistage damage characteristics under cyclic loading. The results of the experimental analysis are as follows.

(1) For the four PVC membrane materials selected in this paper, the multistage cyclic fracture peak strengths of the warp-direction samples are lower than the original tensile strengths, while those for the fill-direction samples are higher than the direct tensile strengths, which is mainly due to the tension difference between the warp and weft yarns during weaving and coating processing. The overall trend of the dissipated energy of the K_1_, K_2_, K_3_, and W_1_ samples is consistent, increasing with the increase in fabric density. Similarly, the fill values are larger than the warp values. According to the analysis of the energy dissipation rate, PVC membrane materials’ use strength should be controlled below a 15% stress level under long-term external force loading.
(2) The loading and unloading curves of the PVC membrane materials are obviously nonlinear, so it is more reasonable to use the linear fitting method to obtain the loading and unloading moduli compared with the approximate equivalent calculation method. For warp knitting materials, the loading and unloading moduli increase with the increase in density, and the values are K_3_ > K_2_ > K_1_. At the same density, the loading and the unloading moduli of the woven material are lower than those of the warp knitting material (W_1_ < K_3_).
(3) Due to the peak strength of multistage cyclic loading being different from that of cyclic loading and unloading, the deformation processes of the warp and fill samples of the four kinds of PVC membrane materials are at different stages at the same cyclic loading times. However, the variation trends of energy and stress show the same law, that is, the total absorbed strain energy, elastic strain energy, and dissipation energy of the warp samples are higher than those of the fill samples under a low load level but lower under a high load level.
(4) Under multistage cyclic loading, the four PVC membrane materials are in the elastic–plastic deformation stage. It is more reasonable to define the damage variable based on the accumulation of plastic deformation than the change in elastic modulus and dissipation energy. Under the same cyclic load peak conditions, the damage levels of the fill samples are higher than those of the warp samples. The damage level of K_3_ is higher than that of W1 for the same density of woven and warp-knitted membrane materials.

## Figures and Tables

**Table 1:** The specifications of samples.

Samples	Warp and Fill Lining Fine- Ness/(Tex)	Warp and Fill Yarn Fine- Ness/(Tex)	Fabric Density/(Yarns/Inch^2^)	Thickness/(mm)	Area Density/(g/m^2^)
K_1′_	111.11	/	9 × 9	0.41	365.10
K_2′_	111.11	/	12 × 12	0.46	436.53
K_3′_	111.11	/	18 × 18	0.51	651.16
W_1′_	/	111.11	18 × 18	0.50	635.73

## Data Availability

The raw data presented in this study are available upon request from the corresponding author.
